# Coral-associated denitrification is seasonally variable and species-specific

**DOI:** 10.1038/s42003-026-11026-w

**Published:** 2026-09-26

**Authors:** Claudia E. L. Hill, Arjen Tilstra, Yusuf C. El-Khaled, Neus Garcias-Bonet, Vivian A. Bonacker, Andres Novoa-Lamprea, Walter A. Rich, Malte Ostendarp, Michael D. Fox, Susana Carvalho, Raquel S. Peixoto, Christian Wild

**Affiliations:** 1 Marine Ecology Department, Faculty of Biology and Chemistry, University of Bremen, Bremen, Germany; 2 Biological and Environmental Sciences and Engineering (BESE) Division, King Abdullah University of Science and Technology (KAUST), Thuwal, Saudi Arabia; 3 Arcadis Nederland B.V, Arnhem, The Netherlands; 4 Groningen Institute for Evolutionary Life Sciences, University of Groningen, Groningen, The Netherlands

**Keywords:** Element cycles, Marine microbiology

## Abstract

Nitrogen (N) plays a critical role in coral growth, but maintaining an N-limited state is essential for coral-algal symbiosis stability. Coral-associated denitrifiers may help regulate excess N, though denitrification in corals remains poorly quantified. To better understand species-specific and environmental effects on the denitrification pathway, we investigated year-long denitrification dynamics in four Red Sea corals, using acetylene inhibition assays alongside physiological and environmental measurements. All species exhibited measurable denitrification activity, ranging from 0 – 0.8 nmol N cm^−2^ h^−1^ for *Stylophora pistillata* and *Acropora* sp., 0 – 0.4 nmol N cm^−2^ h^−1^ for *Millepora dichotoma*, and 0 – 2.0 nmol N cm^−2^ h^− 1^ for Dendrophylliidae indet. Denitrification displayed seasonal patterns, with higher rates in the spring/summer compared to autumn/winter, and we identified temperature, dissolved organic carbon (DOC) and nitrate availability as key environmental drivers. Lastly, we observed up to fivefold higher denitrification rates in the fully heterotrophic azooxanthellate coral Dendrophylliidae indet, than among mixotrophic species harbouring Symbiodiniaceae. Our findings indicate that denitrifiers use both photosynthetically derived and environmental carbon (C), with DOC central in maintaining tight coupling of C and N cycling in coral holobionts. Additionally, denitrification is modulated by environmental conditions, highlighting its vulnerability to environmental change.

## Abstract (web summary)

We explore the influence of seasonal environmental change and the trophic strategy of the coral host on the denitrification activity of four Red Sea corals. We show that denitrification rates are generally higher in the spring/summer compared to the autumn/winter and show the highest rates in the fully heterotrophic coral Dendrophylliidae indet.

## Introduction

Coral reefs are often described as oases in marine deserts, thriving in oligotrophic environments^1,2^. However, globally, the proportion of reefs actually experiencing oligotrophic conditions is low, with 80% of reefs occurring in productive mesotrophic or even eutrophic environments^3^. Despite this, corals are well adapted to withstand nutrient-poor waters, employing a multifaceted approach to efficiently acquire, process and retain nitrogen (N). N is an essential nutrient for corals, supporting protein synthesis, reproduction and photosynthetic efficiency^4–6^. Corals can meet much of their N demand through heterotrophic feeding on N-rich prey and particulate organic matter, if available^7^. Additionally, corals exist as holobionts, living in association with microorganisms such as bacteria, viruses and many other taxa^8,9^. Diazotrophic bacteria form part of this intricate microbial community, playing a crucial role in N-fixation and contributing to the coral’s N budget. Specifically, diazotrophic bacteria reduce atmospheric N_2_ into bioavailable ammonium that can be used by the coral^10–13^ In addition, many stony and soft corals harbour symbiotic dinoflagellates from the family Symbiodiniaceae and are colloquially known as zooxanthellate species^14,15^. The symbionts are capable of taking up nitrate, a process that the coral host itself cannot perform directly as it lacks the appropriate enzymes to reduce it into the bioavailable form of ammonium^16,17^. The symbionts supply the coral host with carbon (C)—rich and N—poor photosynthates^14^. The symbionts also recycle metabolic waste products from the host, such as ammonium^18^, converting these into amino acids and other nitrogenous compounds that are partially translocated to the coral host^19,20^. In contrast, azooxanthellate corals do not host Symbiodiniaceae^21^ and therefore rely solely on heterotrophic feeding and N-fixation for their N supply, without the added benefit of symbiotic N assimilation and C supply.

Under contrasting conditions, corals can be negatively affected when N is available in excess. When more *in hospite* N is available, symbionts allocate more C to their own growth rather than to the coral host, promoting symbiont proliferation and a transition towards parasitism which can trigger coral bleaching^22–29^. The outcome can depend on nutrient stoichiometry, particularly the balance of N and phosphate (P)^6,30,31^. When N enrichment occurs without a corresponding increase in P, the coral-algal symbiosis can break down because the symbionts are starved of P, causing light and heat-induced bleaching^30^. The effects of excess N can also vary by form of N, for example urea-exposed corals recover faster than those exposed to excess nitrate^32^. Additionally, excess ammonium has mixed effects, offering potential benefits to photosynthesis and calcification of corals at moderate concentrations, yet becoming toxic in higher concentrations^33^. Conversely, nitrate may negatively affect both photosynthesis and calcification processes as its conversion into bioavailable ammonium is energetically—costly^34–36^.

One mechanism by which the coral holobiont mitigates excess N is the process of denitrification^6,37,38^. Denitrification is a microbial process where denitrifying microbes within the coral holobiont sequentially reduce nitrate to nitrite, nitric oxide, nitrous oxide and eventually to dinitrogen gas that is released into the atmosphere^39^. This process has received increased attention in recent years, and preliminary insights into denitrification in coral reefs are now emerging. For example, recent studies have revealed that denitrification is an active pathway in multiple stony and soft Red Sea corals^38,40^ stony Cuban corals^41^ and Great Barrier Reef corals^42^. Denitrification has also been identified as an active pathway among several benthic reef substrates such as coral rubble, biogenic rock, turf algae and reef sediment^37,43^. These studies have also revealed that there are differences in denitrification activity among substrates and coral species. Additionally, rates of denitrification and the opposing pathway N_2_ fixation, were found to correlate with algal symbiont density and with each other, leading to speculation that the pathways may have some similarities^38^. For example, Tilstra et al.^38^ hypothesised that the heterotrophic bacteria that govern the two pathways may share a supply of organic C from the algal symbionts. However, although denitrification activity has now been detected broadly, the significance of the pathway in overall N removal in stony corals is debated. For example, Glaze and colleagues^42^ postulated that denitrification has limited importance compared to other N removal pathways like anaerobic ammonium oxidation, whereas Yang and colleagues^44^ found denitrification accounted for ~90% of N_2_ production in stony corals. However, it is important to acknowledge that different techniques have been used to quantify denitrification among existing studies. These include molecular techniques that quantify copy numbers of denitrifying genes such as *nirS*, which can be used as a proxy for denitrification^38,42^ and varying physiological techniques such as tracer experiments (direct)^41,42,44^ and acetylene assays (indirect)^38,40,43^ complicating direct comparisons between studies.

Significant knowledge gaps remain in our understanding of coral-associated denitrification. While previous studies have quantified the denitrification rates of several Red Sea corals, these measurements were conducted under nitrate-enriched conditions to determine denitrification potential^37,38^. Consequently, it remains unclear how denitrification activity responds to natural environmental conditions in the Red Sea. In particular, we do not yet know how coral-associated denitrification varies seasonally. Seasonal cycles in the Red Sea are pronounced, with cooler temperatures (~ 24 °C) and higher nutrient concentrations (e.g., inorganic N) during winter and spring due to vertical mixing, and warmer temperatures (~32 °C) but lower nutrient availability during the stratified summer months^45,46^. The seasonal influence on an alternate N-cycling pathway (N_2_ fixation) has been studied previously, which found significantly higher N_2_ fixation of the reef in spring/summer than autumn/winter^47^. Likewise, in another Red Sea study, higher N_2_ fixation rates were measured in the summer for *Stylophora pistillata* across water depths of 5, 10 and 20 m^48^. Furthermore, previous studies have speculated that there may be a link between denitrification activity and the trophic strategy of the coral host^38,40^, this has also not been directly investigated, and there has yet to be an assessment of denitrification across species with varying trophic strategies.

Considering these knowledge gaps, we asked three key questions: i) What is the influence of seasonal change on coral-associated denitrification rates and which environmental factors drive this process? Secondly, ii) How do internal nutrient dynamics modulate denitrification activity? Lastly, iii) How does the host trophic strategy influence denitrification rates? To assess this, we selected a suite of Red Sea corals that, according to literature, differ in their trophic strategy. We included zooxanthellate corals that have a greater reliance on autotrophy such as *Stylophora pistillata, Acropora* sp. and *Millepora dichotoma*, and an azooxanthellate coral belonging to the family Dendrophylliidae, that is fully heterotrophic^49–52^. We sampled these corals bimonthly (once every two months) over a complete year and measured denitrification rates, assessed various physiological parameters and monitored environmental conditions. We hypothesised that denitrification rates of corals would be lower in winter months compared to summer months as bacterial metabolisms are slowed down by low temperatures^53^. In fact, this pattern in denitrification activity has been observed in seagrass sediments with higher rates measured in summer compared to winter in the central Red Sea^54^. Secondly, we hypothesised that the internal nutrient dynamics of the coral host would influence denitrification activity, given that denitrification activity has been found to correlate with symbiont cell densities^38^, suggesting a link to internal nutrient cycling. Lastly, we anticipated that corals with higher autotrophy would exhibit higher denitrification rates than those that are more heterotrophic. This hypothesis stems from previous studies that suggest that denitrifiers may rely on autotrophically-derived C^38,40^. Filling these knowledge gaps is crucial for comprehending both the natural dynamics of denitrification and the potential impacts of environmental stressors, such as ocean warming and eutrophication, on microbial community structure and function of corals. Furthermore, these findings will shed light on species-specific differences in denitrification and enhance our understanding of how particular species may withstand global changes.

In this study, we present evidence of seasonality in denitrification activity, with higher rates in the spring/summer months compared to autumn/winter. We also found that the denitrification pathway is highly influenced by environmental conditions, yet with species-specific environmental drivers identified. Furthermore, we found that denitrifiers utilise both autotrophically-derived and environmental C to fuel their metabolism, with significantly higher rates of denitrification measured in the heterotrophic coral belonging to the family Dendrophylliidae.

## Results

### Denitrification rates among four Red Sea corals

**Fig. 1:**
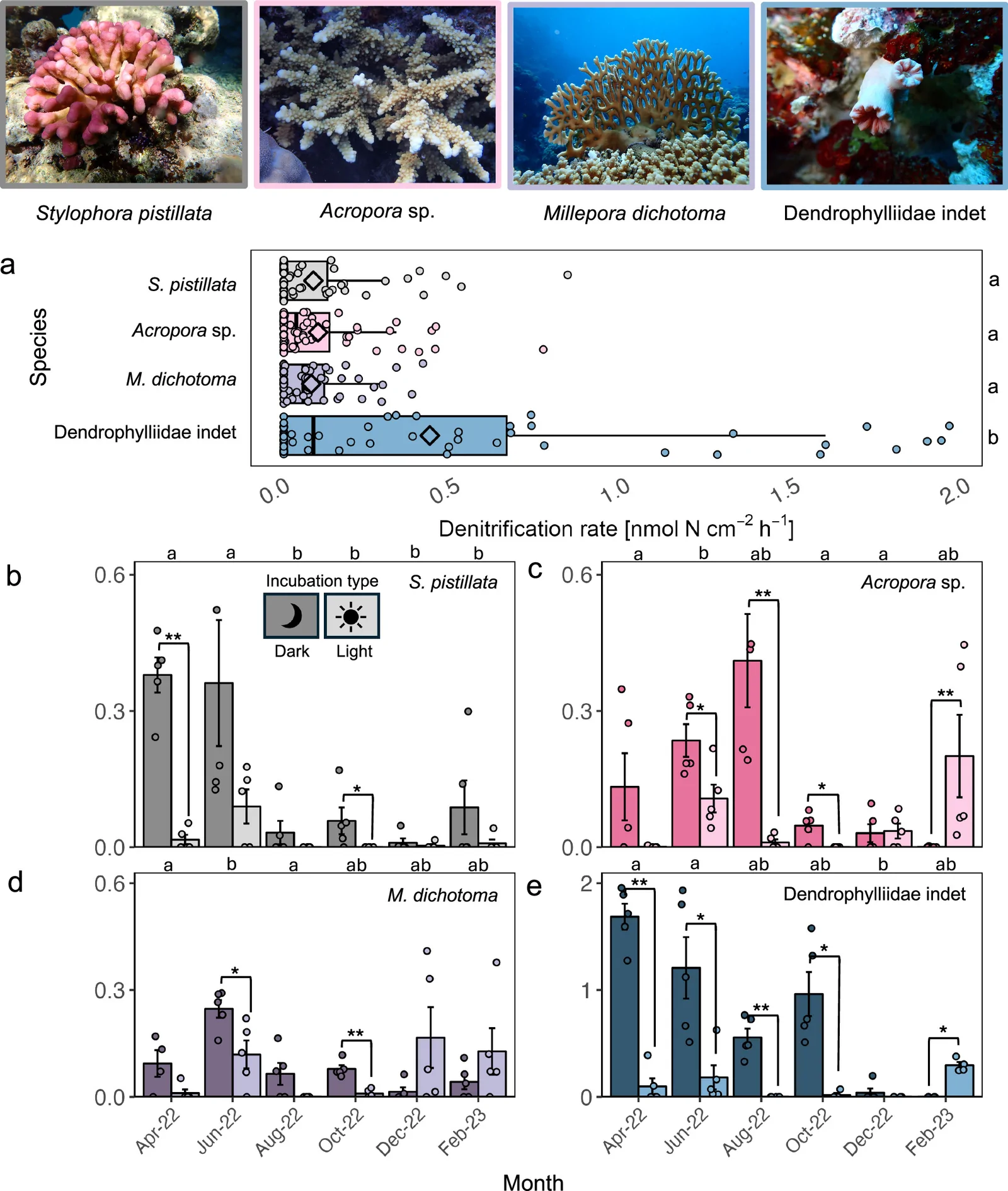
Seasonal denitrification rates of four coral species. **a** Denitrification rates of each *Stylophora pistillata*, *Acropor*a sp., *Millepora dichotoma* and Dendrophylliidae indet, calculated from combined light and dark incubations conducted between April 2022 and February 2023. Diamonds indicate mean values. Differences among species were assessed using a Kruskal-Wallis test followed by a Dunn’s post hoc test. Shared letters indicate no significant differences, whereas different letters indicate significant differences. **b**–**e** Monthly denitrification rates of each species under light and dark conditions. Bars represent the mean ± SE of five biological replicates (*n* = 5); occasionally, fewer than five replicates were available due to sample loss. Note that **e** (Dendrophylliidae indet) uses a different y-axis scale. Differences among months were assessed using a Kruskal-Wallis test, followed by a Dunn’s post hoc test with Bonferroni-adjusted *p* values. Shared letters indicate no significant differences among months, whereas different letters indicate significant differences. Brackets denote significant differences between light and dark incubations within a month (*p *< 0.05 = *, *p* < 0.01 = **). All *p*-values are provided in the Supplementary Materials (Table S1–S3). Photographs are taken by Vivian A. Bonacker.

Denitrification was detected in all four Red Sea coral species (Fig. 1a). Averaged over the year, denitrification rates of the three zooxanthellate species (*S. pistillata*, *Acropora* sp. and *M. dichotoma*) were similar at 0.09 ± 0.16, 0.10 ± 0.16 and 0.08 ± 0.10 nmol N cm^−2^ h^−1^ respectively and therefore did not significantly differ (*p* > 0.05; Fig. 1a). However, the average denitrification rate of the azooxanthellate coral Dendrophylliidae indet was significantly higher than all three zooxanthellate species (*p* < 0.05), being 5-fold higher than both *S. pistillata* and *M. dichotoma* and 4-fold higher than *Acropora* sp. at 0.43 ± 0.61nmol N cm^−2^ h^−1^ (Fig. 1a). *P* values can be found in the Supplementary Material (Table S1).

### Seasonal variation in denitrification rates

Monthly denitrification rates significantly differed across the year in all four species (Kruskal-Wallis; *S. pistillata*: H = 18.2, *p* < 0.01; *Acropora* sp.: H = 14.7, *p* < 0.05; *M. dichotoma*: H = 13.2, *p* < 0.05; Dendrophylliidae indet: H = 14.6, *p* < 0.01), with a trend of higher denitrification activity during the spring and summer months compared to the autumn and winter months (Fig. 1b–e). In *S. pistillata*, denitrification rates were significantly higher in April (0.20 ± 0.20 nmol N cm^−2^ h^−1^) and June (0.23 ± 0.26 nmol N cm^−2^ h^−1^) compared to August (0.02 ± 0.04 nmol N cm^−2^ h^−1^), October (0.03 ± 0.05 nmol N cm^−2^ h^−1^), December (0.01 ± 0.02 nmol N cm^−2^ h^−1^) and February (0.05 ± 0.10 nmol N cm^−2^ h^−1^), all *p* < 0.05 (Fig. 1b). In *Acropora* sp., rates were significantly higher in June (0.17 ± 0.10 nmol N cm^−2^ h^−1^) than in October (0.02 ± 0.03 nmol N cm^−2^ h^−1^) and December (0.03 ± 0.04 nmol N cm^−2^ h^−1^), all *p* < 0.05 (Fig. 1c). In *M. dichotoma*, denitrification rates were significantly higher in June (0.18 ± 0.10 nmol N cm^−2^ h^−1^), than in April (0.05 ± 0.07 nmol N cm^−2^ h^−1^) and August (0.03 ± 0.06 nmol N cm^−2^ h^−1^), all *p* < 0.05 (Fig. 1d). Lastly, in Dendrophylliidae indet, denitrification rates were significantly higher in April (0.89 ± 0.86 nmol N cm^−2^ h^−1^) and June (0.69 ± 0.71 nmol N cm^−2^ h^−1^) than in December (0.02 ± 0.06 nmol N cm^−2^ h^−1^), all *p* < 0.05 (Fig. 1e). *P* values can be found in the Supplementary Material (Table S2).

### Denitrification in the light and dark incubations

Significant differences between denitrification rates measured in light and dark incubations per month were observed in all four species (Fig. 1b–e). Most denitrification rate measurements were significantly higher in the dark than in the light incubations, with this trend observed in 11 out of 13 of the significant pairings identified. More specifically, in *S. pistillata* denitrification rates were 23-fold higher in April in the dark versus the light (Kruskal-Wallis, H = 6.00, p < 0.01) and 0.06 in the dark versus 0 nmol N cm^−2^ h^−1^ in the light in October (Kruskal-Wallis, 5.54, *p* < 0.05; Fig. 1b). In *Acropora* sp., denitrification rates were 2-fold higher in June (Kruskal-Wallis, H = 3.94, *p* < 0.05) and 39-fold higher in August in the dark compared to the light (Kruskal-Wallis, H = 6.99, *p* < 0.01). In October, denitrification rates were 0.05 in the dark versus 0 nmol N cm^−2^ h^−1^ in the light (Kruskal-Wallis, H = 5.54, *p* < 0.05; Fig. 1c). In *M. dichtoma*, denitrification rates were 2-fold higher in June (Kruskal-Wallis, H = 4.81, *p* < 0.05) and 8-fold higher in October in the dark compared to the light incubations (Kruskal-Wallis, H = 6.90, *p* < 0.01; Fig. 1d). Lastly, in Dendrophylliidae indet, rates were 17-fold higher in April (Kruskal-Wallis, H = 6.99, *p* < 0.01), 7-fold higher in June (Kruskal-Wallis, H = 5.77, *p* < 0.05) and 53-fold higher in October in the dark compared to the light (Kruskal-Wallis, H = 6.21, *p* < 0.05), while in August rates were 0.06 in the dark versus 0 nmol N cm^−2^ h^−1^ in the light (Kruskal-Wallis, H = 7.76, *p* < 0.01; Fig. 1e). However, the reverse trend was observed in 2 out of 13 significant pairings, where denitrification was higher in the light incubation compared to the dark incubation. This was observed only during February in *Acropora* sp. where rates were 382-fold higher in the light versus the dark (Kruskal-Wallis, H = 7.26, *p* < 0.01), and Dendrophylliidae indet, where rates were 0.3 in the light versus 0 nmol N cm^−2^ h^−1^ in the dark (Kruskal-Wallis, H = 7.20, *p* < 0.01;Fig. 1c, e). *P* values can be found in the Supplementary Material (Table S3).

### Determining the environmental drivers of denitrification

**Fig. 2:**
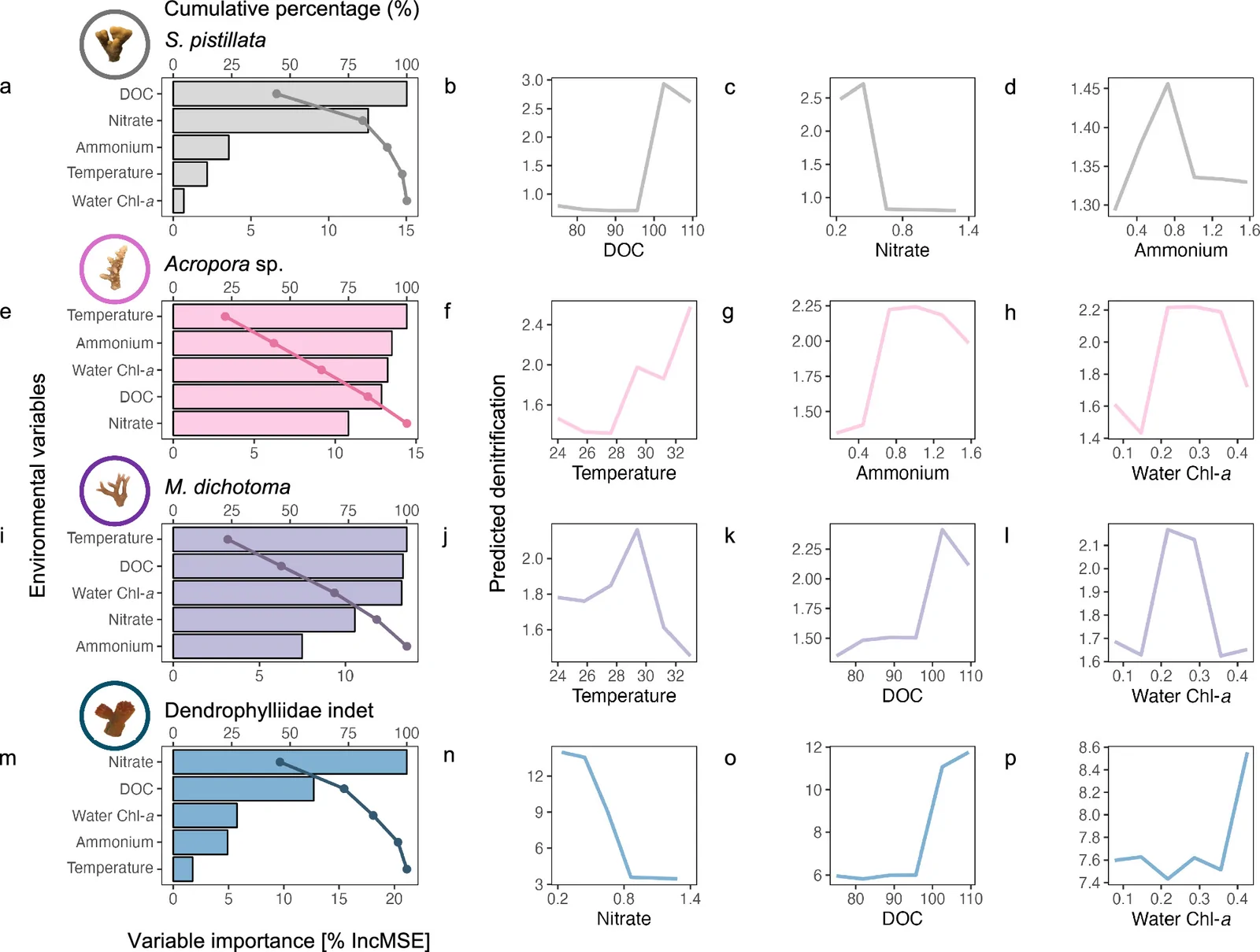
Environmental drivers of denitrification rates in four coral species. *Stylophora pistillata* (**a**–**d**), *Acropora* sp. (**e**–**h**), *Millepora dichotoma* (**i**–**l**) and Dendrophylliidae indet (**m**–**p**). For each species (each row), the leftmost bar plot shows the ranked importance of five environmental variables in explaining denitrifying variation, as identified by random forest analysis. The cumulative contribution of each variable to the overall variation is shown by the auxiliary curve (aligned with the upper x axis). The line graphs for each species are partial dependence plots (PDPs) for the three most influential variables, illustrating how each variable affects denitrification rates. Abbreviations: ‘DOC’ = dissolved organic carbon, ‘IncMSE’ = increase in mean squared error and ‘Water Chl-a’ = water chlorophyll *a*.

The measured environmental variables (nitrate, ammonium, DOC, water chl *a* and temperature) were used in a random forest model, to determine the most influential variables over denitrification rates for each species (Fig. 2). The amount of denitrifying variation that the environmental variables explained, differed between species. For example, the environmental variables explained 12% of denitrifying variation in *S. pistillata*, 57% in *Acropora* sp., 38% in *M. dichtoma* and 50% in Dendrophylliidae indet.

In *S. pistillata*, DOC, nitrate and ammonium were identified as the top three most influential variables over denitrification rates (Fig. 2a). DOC availability was the strongest driver of denitrification in *S. pistillata* (15% IncMSE), with higher levels promoting denitrification (Fig. 2b). Low nitrate availability was the second most influential over denitrification rates in *S. pistillata* (12.5% IncMSE; Figs. 2a, c). Moderate ammonium availability also influenced denitrification rates of *S. pistillata*, yet to a much lesser extent (3.6% IncMSE; Figs. 2a, d). In *Acropora* sp., temperature, ammonium and water chl *a* were most influential over denitrification rates and to similar extents (Fig. 2e). High temperature was the top driver of denitrification in *Acropora* sp. (14.4% IncMSE, Figs. 2e, f), followed by moderate ammonium availability (13.5% IncMSE, Fig. 2e, g) and water chl *a* (13.3% IncMSE; Fig. 2e, h). In *M. dichotoma*, denitrification rates were evenly influenced by moderate temperature (13.6% IncMSE, Fig. 2i, j), high DOC availability (13.3% IncMSE, Fig. 2i, k) and moderate water chl *a* concentrations (13.3% IncMSE; Fig. 2i, l). Lastly, in Dendrophylliidae indet, low nitrate availability was the most influential variable over denitrification rates (21.1% IncMSE, Fig. 2m, n), followed by high DOC availability (12.7% IncMSE; Fig. 2m, o). High water chl *a* concentration (5.8% IncMSE, Fig. 2m, p) was ranked third but influenced denitrification to a lesser extent than the top two variables. Overall, each species was influenced by a unique combination of environmental variables. However, we also identified environmental drivers that are common across multiple species, such as DOC availability and temperature.

Upon repeating the random forest analysis on the light incubation denitrification data, we found that light was not a primary driver of denitrification in any of the species (Fig. S1). However, light was identified as a secondary driver of denitrification activity *S. pistillata*.

### The influence of internal nutrient dynamics on denitrification

**Fig. 3:**
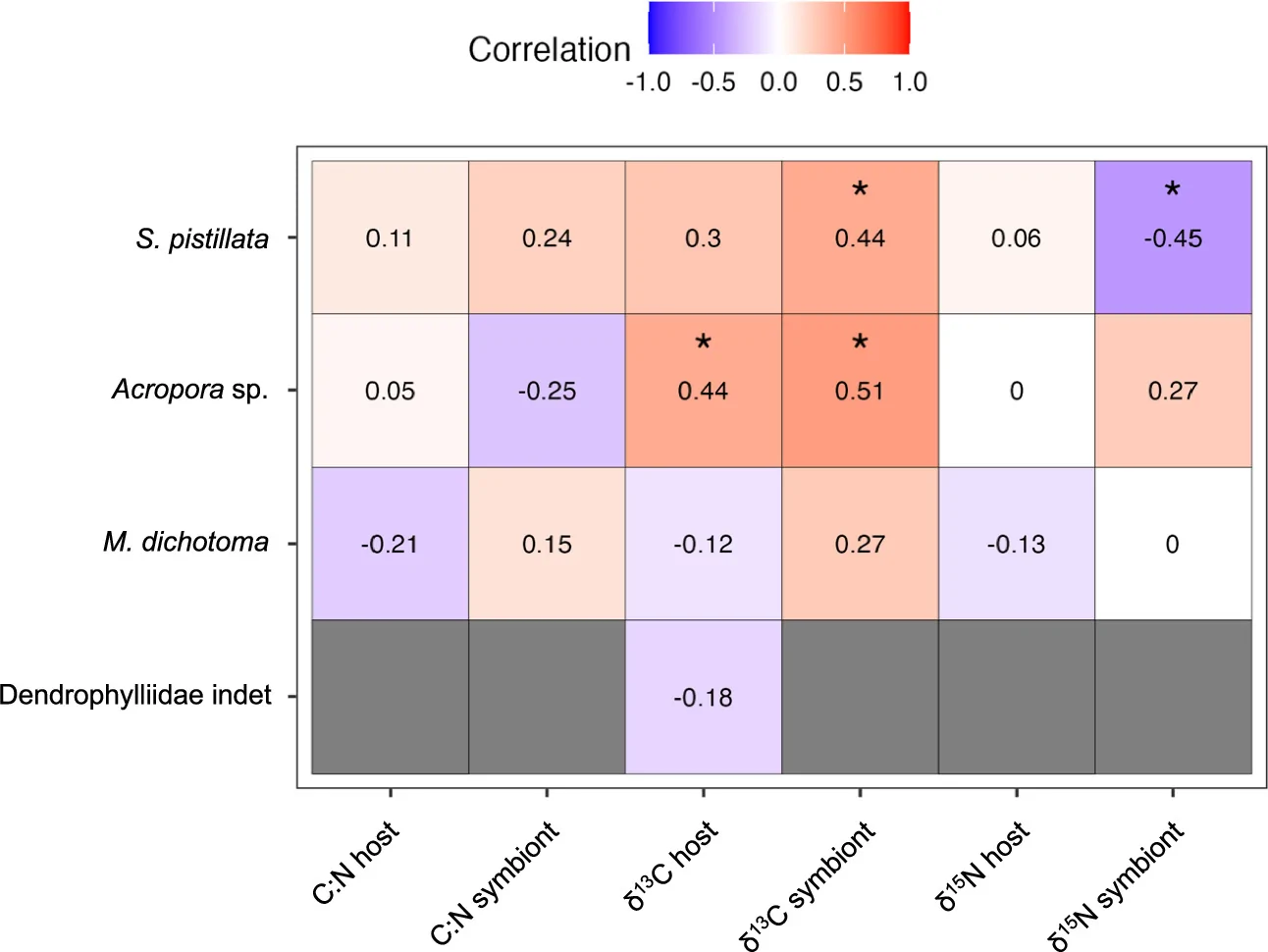
Links between denitrification and biogeochemical signatures. Correlation matrix relating the yearly denitrification rates of *Stylophora pistillata*, *Acropora* sp., *Millepora dichotoma* and Dendrophylliidae indet to their respective biogeochemical signatures. A Spearman’s rank correlation was used, and asterisks (*) denote a significant relationship. In *S. pistillata*, significant relationships were identified between denitrification and δ^13^C symbiont (*p* = 0.03) and δ^15^N symbiont (*p* = 0.02). In *Acropora* sp., significant relationships were identified between denitrification and δ^13^C host (*p* = 0.03) and δ^13^C symbiont (*p* = 0.01).

In *S. pistillata*, we found a significant positive correlation between denitrification rates (0.09 ± 0.16 nmol N cm^−2^ h^−1^) and symbiont δ^13^C (−14.16 ± 0.77‰; *rho*: 0.44; *p* < 0.05), as well as a significant negative correlation with symbiont δ^15^N (2.55 ± 0.54‰; *rho*: − 0.45; *p* < 0.05; Fig. 3). In *Acropora* sp., we found significant positive correlations between denitrification rates (0.10 ± 0.16 nmol N cm^−2^ h^−1^) and host δ^13^C (−15.34 ± 1.03‰; *rho*: 0.44; *p* < 0.05) and symbiont δ^13^C (−14.56 ± 0.48‰; *rho*: 0.51; *p* < 0.05; Fig. 3). In *M. dichotoma*, no significant relationships were detected between denitrification rates and biogeochemical signatures (*p* > 0.05; Fig. 3). Additionally, in Dendrophylliidae indet, no significant relationships were detected between denitrification rates and biogeochemical signatures (*p* > 0.05; Fig. 3). Yet, only one relationship (denitrification and host δ^13^C) could be analysed since symbiont-related parameters were not applicable for this azooxanthellate species, and the other parameters had too few samples for a reliable analysis (Fig. 3).

### Determining the trophic strategy of each coral species

**Fig. 4:**
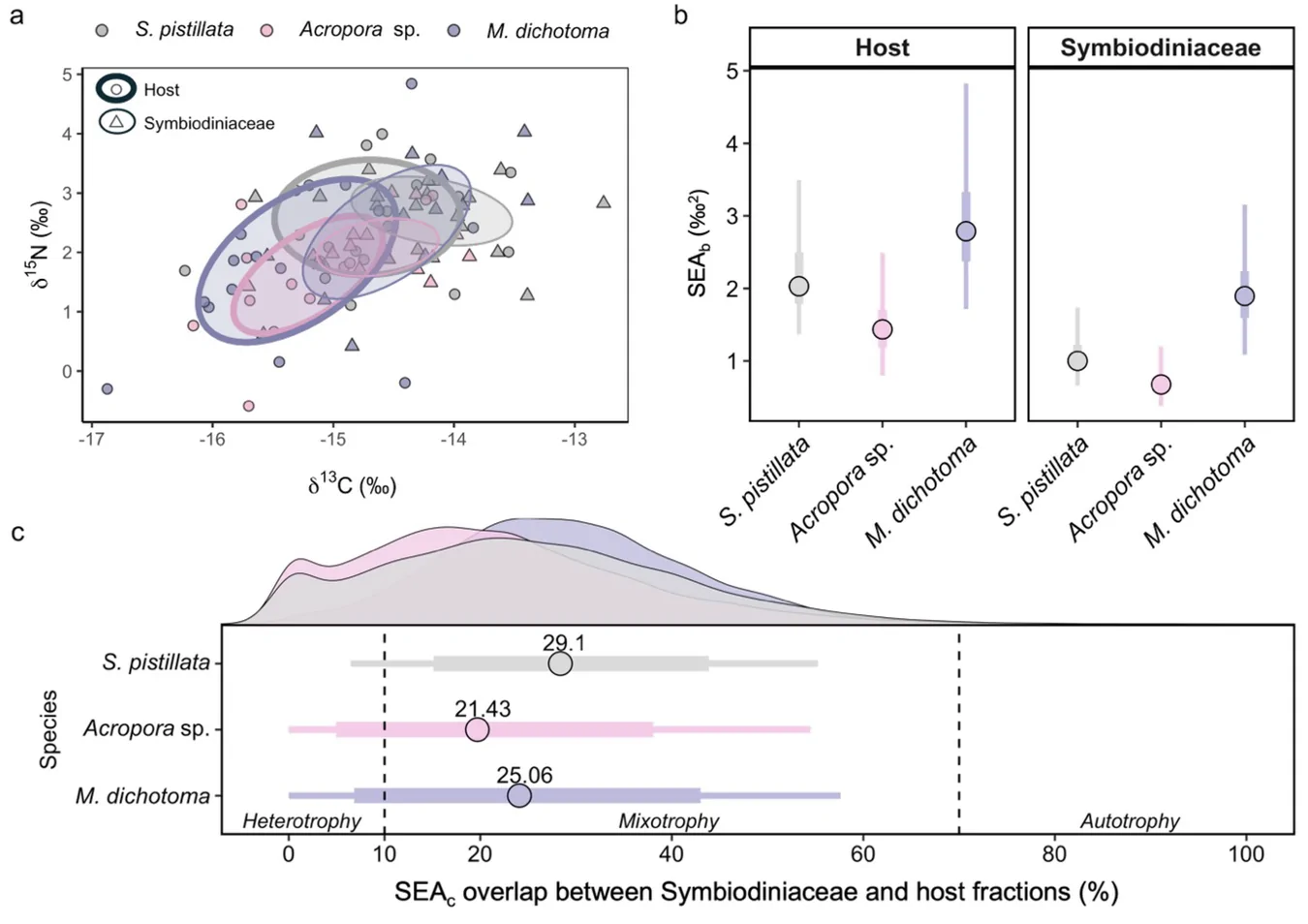
Determination of the trophic strategies of three zooxanthellate corals *Stylophora pistillata*, *Acropora* sp., *Millepora dichotoma.* **a** Standard ellipse areas for the coral host (circles, thick ellipse outline) and Symbiodiniaceae (triangles, thin ellipse outline) representing the core 40% of the isotopic niche. Greater overlap between host and Symbiodiniaceae ellipses indicates higher reliance on autotrophy, while less overlap indicates higher reliance on heterotrophy. **b** Standard ellipse area estimates (SEAb), calculated from posterior sampling of the Bayesian SIBER model, summarised using the posterior mode (coloured circle) along with 50% (thick line) and 95% (thin line) credible intervals. **c** Bootstrapped estimates (*n* = 10,000) of SEAc overlap between the host and symbiont fractions as a proxy for trophic strategy. Coral trophic strategy cutoffs are indicated by dashed lines and labels, according to Conti-Jerpe et al. ^49^. The distribution of the 10,000 overlap estimates are displayed above the plot.

We used Bayesian analysis of isotopic niches^55^ adapted for corals^49,56^ (Fig. 4) combined with Layman metrics of trophic diversity^57^ (Table 1) to quantify and compare the isotopic niches of the three zooxanthellate corals *S. pistillata*, *Acropora* sp. and *M. dichotoma*. The three species showed similarly broad isotopic niche areas (Fig. 4a). However, *M. dichotoma* exhibited the largest Bayesian standard ellipse area (SEAb) of the host fraction (2.87‰^2^, 95% CI: 1.67–4.70), compared to *S. pistillata* (2.15‰^2^, 95% CI: 1.31–3.51) and *Acropora* sp. (1.34‰^2^, 95% CI: 0.86–2.54, as well as the largest Bayesian standard ellipse area (SEAb) of the symbiont fraction (2.34‰^2^, 95% CI: 1.14–3.18), compared to *S. pistillata* (1.05‰^2^, 0.66–1.73) and *Acropora* sp. (0.76‰^2^, CI: 0.38–1.18; Fig. 4b). This was also supported by Layman’s metrics, where *M. dichotoma* had the largest NR, CR and TA (Table 1). However, *M. dichotoma* also had the highest NND and SDNND (Table 1), indicating higher variability between samples. Lastly, when we quantified the relative reliance of heterotrophy versus autotrophy as the percentage overlap between host and symbiont ellipse areas (corrected for sample size (SEAc), the three species all exhibited a mixotrophic feeding strategy, with no marked differences in mean SEAc (Fig. 4c). Although sample sizes were limited, the resampling errors stabilised and converged, rather than spanning the full possible range (0–100%), and patterns were consistent across coral species. Therefore, we believe our interpretations are conservative and robust, despite these constraints.

**Table 1:** Layman metrics^57^ of the three zooxanthellate species *Stylophora pistillata*, *Acropora* sp., and *Millepora dichotoma*

Metric	Description	Interpretation	*S. pistillata*		*Acropora* sp.		*M. dichotoma*	
			Host	Sym	Host	Sym	Host	Sym
δ^15^N range (NR)	Maximum δ^15^N – minimum δ^15^N	Larger NR indicates more trophic diversity.	3.03	2.12	3.55	*1.58*	5.14*	3.61
δ^13^C range (CR)	Maximum δ^13^C - minimum δ^13^C	Larger CR indicates more trophic diversity with varying C sources.	2.70	2.89	1.98	*1.83*	3.03*	2.19
Total Area (TA)	A measure of the amount of niche space occupied	Larger TA indicates a higher extent of trophic diversity. [Can be influenced by extreme/outlier values].	6.71	3.30	3.45	*1.59*	8.01*	5.50
Mean distance to centroid (CD)	Mean Euclidean distance of samples to the δ^13^C - δ^15^N centroid. The centroid is the mean δ^13^C and δ^15^N of samples	Larger CD indicates a higher average degree of trophic diversity. [Less influenced by extreme/outlier values].	1.07	0.72	0.91	*0.57*	1.28*	1.12
Nearest neighbour distance (NND)	Mean of the Euclidean distances between samples in biplot space	Small NND indicates similar trophic ecologies between samples.	0.53	0.34	0.42	*0.27*	0.56*	0.46
Standard deviation (SD) of NND	Evenness of spacing between samples	Small SDNND indicates a more even distribution.	0.34	0.26	0.35	*0.16*	0.53*	0.28

Description and interpretation guidelines are adapted from Layman et al.^57^. Values are shown for the host and symbiont (Sym) fraction. The lowest value of the sample groups is italicised, while the highest value is indicated with an asterisk (*).

The trophic strategy of Dendrophylliidae indet could not be quantified because this analysis requires isotope data from both host and symbiont fractions, and since Dendrophylliidae indet lacks symbionts, the method was not applicable. Though, as an azooxanthellate coral, its feeding mode is already known to be entirely heterotrophic. We were, however, able to measure host parameters for Dendrophylliidae indet and the mean host δ^13^C was −24.01 ± 2.08‰, which was higher than the mean host δ^13^C of *S. pistillata* (−14.72 ± 0.75‰), *Acropora* sp. (−15.21 ± 0.60‰), and *M. dichotoma* (−15.3 ± 0.80‰; Fig. S2). Unfortunately, host δ^15^N could not be reliably quantified because too few samples remained following data cleaning steps (detailed in the Data analyses section).

## Discussion

Among four coral species, we observed seasonal patterns in denitrification activity, demonstrating how the N-cycling pathway is highly influenced by environmental conditions. In addition, denitrification rates were influenced by the internal nutrient dynamics of the coral holobiont and may be linked to the trophic strategy of the coral host.

The denitrification rates measured here are comparable to the ranges presented in previous studies on Red Sea corals, once appropriate conversions are applied (Fig. 1a)^38^. Rates also significantly varied throughout the year for all four species, with a general seasonal trend of higher rates in the spring/summer compared to the autumn/winter months of the year (Fig. 1b). The effect of seasonality on denitrification has not been investigated before in corals, but studies on other systems such as estuaries and marshlands have reported similar seasonal patterns to our study, finding higher denitrification rates and, in addition, higher denitrifier diversity in spring^58,59^. The seasonal trend observed in these former studies and our own can be explained by the sensitivity of the denitrification pathway to environmental conditions that fluctuate over a year (Fig. S4).

Temperature emerged as the primary driver of denitrification in *Acropora* sp., and *M. dichotoma*, though its influence differed between the two species. In *Acropora* sp., denitrification rates increased linearly with temperature. This relationship reflects the general principle of higher temperatures stimulating microbial metabolism and activity^53^. The same pattern was observed in Red Sea seagrass sediments^54^. Additionally, among corals, elevated temperatures increase the activity of diazotrophs that govern N_2_ fixation^60^ introducing more *in hospite* N available for denitrification. Furthermore, a recent study by Rädecker et al.^61^ demonstrated that under high temperatures, the coral catabolises amino acids, again introducing more *in hospite* N available for denitrification. For *M. dichotoma*, however, denitrification rates peaked at moderate temperatures and decreased at higher temperatures. These differences may reflect the response (and presence of) species-specific denitrifying microbiomes, as has been found between corals in previous work^62^. While there is no literature directly comparing the denitrifying microbial communities between *Acropora* sp. and *M. dichotoma*, studies reveal that *Acropora* sp. hosts a greater bacterial diversity than *M. dichotoma*^63^, indicating a potential for differences.

High DOC availability was identified as the top driver of denitrification in *S. pistillata*, and the second driver in *M. dichotoma* and Dendrophylliidae indet. The positive relationship between DOC and denitrification has been widely observed in other environments i.e., sediments and freshwater systems^64–67^ and is attributed to the role of DOC as a key element supporting heterotrophic microbial growth and activity^68^. Therefore, our study provides evidence that denitrifiers in coral holobionts are also capable of utilising environmental DOC as a source of C, and may not solely rely on symbiont derived C. One study investigating the influence of DOC on octocoral denitrification reported contrasting results, where excess DOC (supplied as glucose) reduced denitrifier abundance by an order of magnitude in *Xenia umbellata*, but had no effect on *Pinnigorgia flava*^69^. This discrepancy may be due to variations in the composition and concentration of DOC^69^.

Against expectations, low nitrate availability was another key driver of denitrification. Nitrate is essential to the denitrification process, acting as an electron acceptor for denitrifying bacteria, which sequentially reduces it to dinitrogen gas^70^. Consequently, one may expect that the more available nitrate, the more is taken up by the coral symbionts (among zooxanthellate corals) and the higher the denitrification rates. However, the opposite relationship was apparent in our study, with denitrification rates linked to low nitrate availability (Fig. 2). This may simply be because denitrification activity on an ecosystem scale is sufficiently high to reduce nitrate concentrations in the surrounding water column. Alternatively, a study by El-Khaled et al.^37^ demonstrated that N cycling is nuanced, with opposing pathways like N_2_ fixation and denitrification increasing together under higher environmental N. In the oligotrophic Red Sea, where nitrate stays low year-round (0.2–1.3 μmol L^−1^; Fig. S4b), N_2_ fixation may rise in response to low N while denitrification simultaneously increases. This suggests that denitrification becomes dominant only at higher nitrate concentrations, whereas at lower concentrations it may co-occur with N_2_ fixation as previously found^37^. To fully explain this finding, however, further investigation into denitrification and N_2_ fixation activity in response to a range of DIN concentrations is required.

Furthermore, we found that the influence of light on denitrification activity was highly nuanced. For example, when comparing the rates between the dark and light incubations, denitrification was significantly higher under dark conditions, with only a few cases where this pattern was reversed (Fig. 1b–e). This likely reflects reduced oxygen availability in the dark where the absence of photosynthesis with continued respiration creates conditions that favour denitrification by anaerobic microbes^71^. Therefore, our study provides evidence that denitrification is a more active pathway under low oxygen conditions, aligning with findings of former studies^43^. However, although high light was not a major driver of denitrification across three of the four species, it emerged as a secondary driver of denitrification relative to other environmental factors in *S. pistillata* (Fig. S1). This may be explained by the greater availability of organic C fuelling denitrifying activity, produced through photosynthesis under light conditions. Taken together, these findings suggest that light does not directly regulate denitrification, but instead indirectly influences the coral’s metabolic environment in ways that can promote or suppress denitrification activity.

While the random forest analysis highlighted key environmental drivers of denitrification, a portion of denitrifying variability remained unexplained by the environmental parameters in our study. For example, 12% of denitrifying variation was explained by environmental conditions for *S. pistillata*, 57% for *Acropora* sp., 38% for *M. dichotoma* and 50% for Dendrophylliidae indet (Fig. 2), emphasising the potential influence of additional factors beyond the scope of our study.

By correlating denitrification rates with species-specific biogeochemical signatures, we found a significant negative correlation between denitrification rates and symbiont δ^15^N in *S. pistillata* (Fig. 3). This is an indication that denitrification may enhance or maintain internal N-limitation within the coral holobiont, as expected^6^. However, this relationship was not observed for the other zooxanthellate species *Acropora* sp., and *M. dichotoma*, challenging the functional role of denitrification in these species, which we expand upon later. Furthermore, we found a significant positive relationship between denitrification rates and symbiont δ^13^C in *Acropora* sp. and *S. pistillata*, and an additional significant positive correlation between denitrification and host δ^13^C in *Acropora* sp. (Fig. 3). These findings provide evidence that denitrifiers utilise autotrophically derived-C, as seen in former studies^38,40,69^. However, we of course also provide evidence that denitrifiers utilise environmental-DOC (Fig. 2). Naturally, this raises the question of how host trophic strategy might further influence denitrification activity.

We also examined the influence of host trophic strategy on denitrification rates. Unexpectedly, denitrification rates of the fully heterotrophic coral Dendrophylliidae indet were significantly higher than those of all other species in our study, being 5-fold higher than both *S. pistillata* and *M. dichotoma* and 4-fold higher than *Acropora* sp. (Fig. 1a). This result contradicts our initial hypothesis where we predicted higher denitrification rates in the more autotrophic species based on findings from former studies^38,40,69^. Therefore, our findings suggest that environmental C may even fuel denitrification at a faster rate than photosynthetic C, given the exceptionally high rates measured in Dendrophylliidae indet. However, such high rates may also be attributed to other factors beyond the C source. For example, Dendrophylliidae indet may host a more diverse and efficient denitrifying community^62^. We also need to measure the denitrification activity of additional heterotrophic species to see whether this finding is unique to Dendrophylliidae indet or applies broadly to heterotrophic species. Furthermore, since our species were all mixotrophic (Fig. 4), we could not assess the denitrification activity in more autotrophic species and would suggest future work to include a species that exhibits a greater reliance on autotrophy. However, since we had to pool the isotopic data over the year due to limited sample sizes, we may have missed seasonal shifts towards greater autotrophy in our species. Therefore, we would recommend higher sample sizes at each seasonal time point.

Furthermore, by mitigating excess N, denitrification is proposed to sustain N-limitation within the coral holobiont, which is critical to the stability of the coral-algal symbiosis^6^. However, the occurrence of denitrification in an azooxanthellate coral challenges this proposed functional role. Our findings suggest that denitrification is not an adaptive trait among azooxanthellate corals, but rather a passive, opportunistic response to a suite of environmental conditions that favour its activity. This interpretation aligns with previous research on denitrification in octocorals^69^ and tropical scleractinian corals^42^.

Having addressed the specific research questions of our study, it is important to interpret these results in the context of the Red Sea’s unique characteristics for a broader perspective of their ecological relevance. The Red Sea is one of the warmest and saltiest seas on Earth, exhibiting strong temporal and spatial gradients^45^. Spatially, the temperature, nutrient availability and chl *a* concentration differs between the north and south of the Red Sea, with the highest temperature, DIN and chl *a* occurring in the south, and decreasing northwards^45,72^, while the nutrient availability in the shallow zone is very low and increases with depth. Our study was conducted in shallow waters of the central Red Sea where environmental conditions were found to have a strong influence over denitrification activity. Broadly, temperature and nutrient availability emerged as key drivers of denitrification. Based on our findings, we anticipate that corals of the same species may exhibit spatial variation in denitrification activity across the Red Sea in response to environmental conditions. For example, denitrification activity in *Acropora* sp. may increase southwards as temperature and DIN increases. However, caution should be taken when extrapolating our seasonal findings beyond the Red Sea, as seasonal dynamics differ among coral reef regions worldwide. Some regions experience weak seasonality such as the equatorial Indo-Pacific. Therefore, it is likely that the same spike in denitrification activity in the spring/summer seasons as measured in our study, may not be seen globally.

## Conclusions

Our study shows that the denitrification pathway is seasonally variable in corals, exhibiting generally higher rates in the spring and summer months compared to the autumn and winter months (Fig. 1b), which can be explained by the high sensitivity of denitrification to environmental conditions (Fig. 2). Our study identified species-specific environmental drivers of denitrification (Fig. 2). The top driver in *S. pistillata* was DOC, providing evidence that denitrifiers do not exclusively rely on photosynthetic C for energy (Fig. 2). For *Acropora* sp., and *M. dichotoma*, the top driver was temperature, although its influence differed between the two species (Fig. 2). In *Acropora* sp., we found a linear relationship between denitrification and temperature, whereas in *M. dichotoma*, denitrification activity increased with temperature until an upper threshold, beyond which it declined (Fig. 2). For Dendrophylliidae indet, we unexpectedly identified low nitrate availability as a driver of denitrification (Fig. 2), yet this is likely the effect not the cause of high denitrification activity, or this is due to the co-occurrence of denitrification with N_2_ fixation when nitrate levels are low. Our study also showed the nuanced role of light on denitrification, with findings suggesting that light influences the coral’s metabolic environment in ways that can indirectly promote or reduce denitrification activity. Furthermore, our study demonstrated that denitrification is also influenced by the internal nutrient dynamics of the coral holobiont, as the positive relationship between denitrification and host ∂^13^C in *S. pistillata*, and both host and symbiont ∂^13^C in *Acropora* sp. (Fig. 3), suggest that denitrifiers utilise symbiont-derived C as shown in former studies^73^. Lastly, we found that denitrification rates were affected by host trophic strategy, finding significantly higher denitrification in the azooxanthellate, fully heterotrophic coral Dendrophylliidae indet, being 5-fold higher than both *S. pistillata* and *M. dichotoma* and 4-fold higher than *Acropora* sp. (Fig. 1a) that we determined were all mixotrophic (Fig. 4). This suggests that environmental C may even fuel denitrification at a faster rate than photosynthetic C, or points to the influence of distinct denitrifying communities that may exhibit different rates. However, the exceptionally high denitrification rates measured in the azooxanthellate species challenge the functional significance of N removal, since no coral-algal symbiosis is present to necessitate such regulation. Therefore, we suspect that denitrification may play a more passive role than previously suspected among Red Sea corals, reinforcing findings from coral-associated denitrification research in other geographic regions^42^ and on octocorals^69^. Overall, our study established a valuable baseline for seasonal denitrification rates in the Red Sea, and an understanding of its environmental drivers across four species. This foundation offers a springboard for future research to explore the influences of environmental change on N cycling in greater detail.

## Material and methods

### Collection of corals

We carried out coral collections in the central Red Sea at the ‘Al Fahal Reef’, or also known as ‘The Coral Probiotics Village’ (22.30518N, 38.96468E), a mid-shore reef located 15 km offshore from the King Abdullah University of Science and Technology (KAUST), Saudi Arabia^74^. The sampling area is shallow, with a maximum water depth of 10 m. We identified four species of Red Sea corals that differ in their trophic strategy, including three zooxanthellate species *S. pistillata*, *Acropora* sp., and *M. dichotoma* that exhibit mixotrophic feeding and an azooxanthellate coral belonging to Dendrophylliidae that has a fully heterotrophic lifestyle^49–52^. We sampled five separate colonies (n = 5) of each species using SCUBA between 1 and 5 m water depth, every second month over a one-year timespan, generating six timepoints i.e., April 2022, June 2022, August 2022, October 2022, December 2022, and February 2023. We consistently carried out sampling in the first two weeks of every other month, sampling *M. dichotoma* and *Acropora* sp. in the first week, and *S. pistillata* and Dendrophylliidae indet in the second week. From each colony, we cut three fragments using pliers and placed them into labelled sampling bags, filled with seawater. Out of the three fragments, we used two for incubations (~5 cm length) and one for isotope and elemental analysis (~5 cm length). In the case of Dendrophylliidae indet, colonies were too small to sample multiple fragments from, so instead, we sampled 15 polyps bimonthly. On the boat, we stored the fragments for incubations in recirculation aquaria filled with seawater from the sampling site, equipped with an air pump to maintain water circulation and oxygen availability. We placed all aquaria in the shade to prevent heat/light stress during transport, and we kept the fragments for physiological assessments on ice and later stored them at −20 °C in the lab.

The collections were conducted as part of a collaboration between KAUST and the University of Bremen, in which a subset of the dataset (isotopic and elemental data) for two species (*S. pistillata* and *M. dichotoma*) was analysed separately^75^.

### Quantification of denitrification rates

On the same day as sampling, we quantified denitrification rates via acetylene blockage/inhibition assays. This indirect method has been successfully applied to investigate coral reef-associated denitrification activities^76^. Acetylene blocks the activity of the enzyme nitrous oxide (N_2_O) reductase within the denitrification pathway, leading to the accumulation of N_2_O which can be used as a proxy for the relative activity of denitrification of the coral holobiont^76–79^. The efficacy of acetylene assays has been validated in previous work which used both acetylene assays and *nirS* gene copy numbers to quantify denitrification^38^. The observed patterns of *nirS* gene abundance corresponded closely with the denitrification rates measured using the acetylene method, providing evidence that the acetylene method is accurate and can be relied upon^38^.

To set up the acetylene assays, we secured each coral fragment to a stand using rubber bands and placed them inside a gas-tight glass beaker (Fig. S3). We specially adapted the beakers for acetylene assays by having an 8 mm hole drilled into the lid. The hole was sealed with a gas-tight rubber stopper, and a hypodermic needle (hypodermic needle with polypropylene hub 30 G × 3/4 in., Tyco Healthcare group, MonojectTM) was permanently inserted through the stopper into the jar. On the outside of the jar, the needle hub was attached to a 2-way stopcock with a Luer lock connection (two-way stopcock, BraunTM DiscofixTM) where a gas syringe (50 ml gastight syringe model 1050 TLL PTFE Luer Lock, Hamilton) could be later fitted when required, for gas samples to be taken (Fig. S3). We filled each beaker with seawater (taken from the sampling site the same morning) to 80% of its capacity, leaving a 20% headspace. We then replaced 10% of the seawater volume with acetylene-enriched seawater, and likewise, replaced 10% of the beaker headspace with acetylene gas^79^ (Fig. S3). We made acetylene gas freshly on the same day prior to the incubations (detailed in the Supplementary). For the incubations, we secured corals to a stand in gas-tight glass beakers filled with site-collected temperature-controlled seawater. We placed beakers into water baths equipped with thermostats (3613 aquarium heater. 75 W 220-240 V; EHEIM GmbH and Co.KG) and temperature controllers (Schego Temperature Controller TRD, max. 1000 W) that heated the water to the corresponding in situ temperature per sampling month. The water bath was then placed on top of magnetic stir plates which powered magnetic stir bars in the base of each beaker to ensure adequate water circulation (~220 rpm). We incubated corals for 12 h in the dark followed by 12 h in the light. During the light incubation, we supplied light at the same intensity as the in situ conditions of the respective sampling month (see Table S4 and Fig. S4h). We achieved these irradiances by adjusting the light intensity dial of the dimmable 165 W full-spectrum LED aquarium lights (Galaxyhydro) and using a LI-193 Spherical Underwater Quantum Sensor (LI-COR). We used new fragments for the following light incubation to minimise the potential impact of stress on the coral’s denitrification rates. We included four control beakers containing no corals in each incubation run to account for potential background denitrification activity in the seawater. We took gas samples (3 ml) from the beaker headspace at the beginning (T0) and end (T12) of each incubation, using a gas syringe (50 ml gastight syringe model 1050 TLL PTFE Luer Lock, Hamilton) and stored the samples in gas-tight vials until further measurement.

Gas samples generated from the acetylene assays are typically measured using a gas chromatograph (GC) fitted with an electron capture detector (ECD). However, in our study, we measured the gas samples using a N_2_O microsensor (custom-made, Unisense). Although a GC with ECD was tested, it was not used as microsensors outperformed it in several aspects. For example, the electrochemical microsensor has high sensitivity (detection limit: 25 nM) and a higher throughput compared to GC-based methods. We connected the microsensor to a multi-channel (fx-6 UniAmp multi-channel 110394, Unisense) and calibrated it every day prior to usage with a two-point calibration curve consisting of a low point (ambient air: 0.009 μmol L^−1^) and a high point (a known standard: 0.575 μmol L^−1^) at a consistent room temperature (21 °C) and pressure (1 bar). To record and visualise measurements, we synced the N_2_O microsensor with the Sensor Trace Suite software (v.1.13) on a computer desktop. Further technical details about the microsensor and the steps to use the microsensor and normalise the data can be found in the supplementary material.

While the acetylene inhibition technique has historically been used to quantify denitrification rates^38,40,43^, it has several limitations^76,80^. For instance, incomplete inhibition of N_2_O reductase occurs, meaning that N_2_ is produced as normal instead of accumulating as N_2_O, causing an underestimation of denitrification rates^76^. Further underestimation of denitrification may arise from the inhibition effect of acetylene on the nitrification pathway^81^. Nitrification is the preceding step in the N-cycle that provides nitrate as a substrate for denitrification. Therefore, denitrification rates may be underestimated due to substrate limitation^80–82^. Another common technique is to run ^15^N tracer incubations^42^, which measures the actual N_2_ production directly. The benefits of this technique include the ability to distinguish between pathways and the lack of an inhibitory effect on related pathways. However, in our case, using the acetylene inhibition technique was more suitable, as it allowed us to measure denitrification activity under ambient conditions, without artificially enriching with ^15^N-labelled substrates.

### Elemental and isotopic analysis of carbon and nitrogen

Firstly, we blasted the tissue off the coral skeleton. To do so, we held the fragment within a sterile clear sampling bag (Whirl-pack sample bag) and used an airbrush (model S68 with dual action siphon feed, Master Airbrush) to remove the tissue with high-pressure air and MilliQ water. We always sterilised the airbrush with 70% ethanol and rinsed it with MilliQ water between samples. We then stored the tissue slurries in Falcon tubes at −20 °C and defrosted them in prior to the next stage. Once samples had defrosted, we separated the tissue slurry into host and symbiont fractions via centrifugation (2.5 min at 500 × *g*) and washed and resuspended the symbiont pellet in 3 ml of MilliQ until it was clean (i.e., free from white residue), which typically required three washes. Following this, we filtered each fraction through 0.7 µm GF/F filters (fitted to a vacuum assembly), treated to remove skeletal contaminants (2 ml × 1N HCl), rinsed with MilliQ, and immediately dried the samples at 60 °C for 48 h. We then scraped the dried mass into tin cups and weighed and analysed the samples for δ^13^C, δ^15^N and mass percent C and N via EA-IRMS at the Natural History Museum, Berlin, using a Flash 1112 EA and Thermo Scientific Delta V IRMS (Berlin, Germany). We report the isotopic data using the conventional delta notations (δ) and expressed in ‰ relative to the international standards (Vienna Pee Dee Belemnite) for δ^13^C (0.01118) and atmospheric N_2_ for δ^15^N (0.00368)^83^. Within-run standard deviations (SD) of the standards were < 0.15 per mil (‰) for δ^13^C and δ^15^N and the SD of replicate measurements of the lab standard are < 3% of the concentration analysed.

### Measurement of environmental parameters

Twenty-four hours before sampling for nutrients, we acid-washed all water sampling equipment in a 4% HCl bath to minimise potential contaminations. On the same day as coral fragments were collected, we collected water samples from the average study site depth (~3–5 m depth) using 2 × 5 L Niskin bottles. On the boat, we aliquoted water for separate nutrient measurements into Falcon tubes. Immediately on the boat, water aliquots for nitrite and nitrate were filtered (0.22 μM Millex®-GV), yet we performed no immediate filtration steps for ammonium, DOC and chl *a* (stored in opaque bottles). We kept all water samples on ice during transport and then stored samples for nitrite, nitrate and ammonium at −20 °C, and stored samples for chl *a* and DOC analysis at +4 °C.

For the measurement of nitrate and nitrite, we analysed samples with a segmented flow analyser (Model AA3 HR, SEAL Analytical IC). We performed a calibration prior to every run and accepted upon the criteria that *R*^2^ > 0.99. To prepare the calibration standards, we used a ready-made stock standard of 1000 ppm nitrite and nitrate to create low (5, 20, 50 and 100 ppb) and high (100, 200, 500, 1000 ppb) standards. The instrument detection limit for nitrate was 0.0322 μmol L^−1^ and 0.0217 μmol L^−1^ for nitrite. For the measurement of ammonium, we analysed samples using a fluorometer (Turner Designs, Trilogy Fluorometer) via Orthophthaldialdehyde (OPA) derivatisation. We performed a calibration prior to every run and accepted upon the criteria that *R*^2^ > 0.99. We prepared a mother solution of ammonium chloride to make standards of 0.0, 0.03, 0.05, 0.1, 0.15, 0.2, 0.25, 0.3 µmol L^−1^. The detection limit of the instrument was 0.058 μmol L^−1^. For DOC analysis, we filtered 500 ml of water through 0.7 µm GF/F filters (pre-combusted at 450 °C for 4.5 h). We divided the filtrate into sterile amber glass vials, that we then acidified with 0.1 ml of 85% phosphoric acid to prevent bacterial activity and analysed them on a TOC Analyser (TOC-L, Total Organic Carbon Analyser, Shimadzu, Kyoto, Japan). We performed a calibration before each run using a standard addition curve of Potassium Hydrogen Phthalate (0; 33; 25; 50; 62; 83; 100; 125; 167; 250; 500 μmol C L^−1^). We prepared internal controls using Consensus Reference Materials (CRM; Batch 12; 2012; DOC: 42–45 μmol L^−1^) provided by DA Hansell and W Chen (University of Miami). The average analytical variation of the instrument was < 3.5% for DOC based on 5–7 injections per sample.

On the same day as collection, we filtered water samples for chl *a* (2 L in duplicates) through 0.7 µm GF/F filters and then stored them at −80 °C until further processing. We prepared samples for measurement adapting a protocol from Erar and Collins^84^ and a protocol used in the California Operative Oceanic Fisheries Investigations. In brief, we soaked the filters in 10 ml of 90% acetone, vortexed, sonicated in an ice bath and stored them at 4 °C overnight in the dark. The following morning, we repeated the sonication and vortexing steps twice more, and then centrifuged the samples at 2500 rpm at 4 °C for 10 min. Next, we measured the samples fluorometrically (Turner Designs, Trilogy Fluorometer) on a fluorometer fitted with a chl *a* module (Turner Designs, Wide-Chlorophyll *a* Acidification Module). We calibrated the instrument prior to use with standards of 0.5, 1.0, 2.5, 5.0, 10.0, 20.0, 100.0 and 200.0 µg L^−1^ which generated a calibration curve of *R*^2^ > 0.99. Following this, we validated the instrument calibration with two solid secondary standards adjusted to 2.5 and 20 µg L^−1^ (Turner Designs, Adjustable Solid Secondary Standard - Red) and ran ‘blanks’ of 90% acetone at the beginning, and after every few samples.

Lastly, we measured seawater temperature continuously throughout the sampling period using an Onset Hobo pendant temperature logger (Pro v2 data logger U22-001) deployed at the reef. We deployed the logger at 1–2 m depth, and measured temperature at 10 min intervals throughout the sampling period April 2022–February 2023. We wrapped the logger in white electrical tape to minimise solar bias and achieve better measurement accuracy^85^. For light intensity, we deployed an additional Onset Hobo pendant logger with a light sensor (UA-002-64; spectral detection range of 150– 1200 nm) at 2 m depth and measured the light at 5 min intervals. We affixed the logger to a flat tile secured to a metal stake, to ensure the sensor was oriented directly towards the sunlight. Each sampling month, the data were downloaded and converted from lux to photosynthetic active radiation (PAR) using a conversion factor of 51.8, as used in previous literature^86^, where 1 μmol quanta m^−2^ s^−1^ = 51.8 lux. Data from only the first 3 days of deployment were included, before biofouling compromised data accuracy. Therefore, the mean peak irradiance was calculated from the first three days between 11 a.m. and 1 p.m. All in situ environmental parameters can be found in the Supplementary Material (Fig. S4).

### Data analyses

We assessed denitrification data for statistically significant differences between species, between months per species and between light and dark incubations per month and species. All denitrification rate data were tested for normality via Shapiro-wilk tests, revealing that data were not normally distributed, even following transformations. Therefore, the non-parametric Kruskal-Wallis test was used followed by a Dunn’s test with Bonferroni p-value adjustment for post-hoc analysis.

Secondly, we employed a random forest model to identify key environmental variables that may influence denitrification rates across different coral species. For this, we used the combined denitrification rates of the light and dark incubations as this integrates diel variability and therefore more accurately represents in situ ecological functioning. The random forest regression model consisted of 500 trees, with 3 variables tried at each split. The variable importance for predicting denitrification was determined by the mean squared error (%MSE), where a high %MSE indicates that the variable is important, as if it were removed, the model’s error would significantly increase. As a follow-up to the random forest analysis, we plotted partial dependence plots (PDPs) for the top 3 most influential parameters per species. This provided insight into how these parameters interacted with denitrification, by displaying how changes in one parameter influences the predicted outcome, while averaging the influence of all other parameters. To determine the influence of light intensity on denitrification rates, the random forest analysis was repeated on the light incubation denitrification rates.

Next, we correlated the denitrification rates of each species with its measured biogeochemical signatures, including isotope (δ^15^N and δ^13^C) and elemental data (C:N) of both the host and symbiont. For this, we used a non-parametric Spearman rank correlation, as data were not normally distributed following Shapiro-wilk testing. Data were pooled across the sampling year and cleaned prior to analysis. This included removing samples below the instrument’s detection limit of 0.015 mg, followed by identifying and excluding statistical outliers.

Lastly, the baseline trophic strategies and niche widths for the three zooxanthellate corals (*S. pistillata, Acropora* sp., and *M. dichotoma*) were estimated following the approach of Jackson et al.^55^ adapted for corals by Conti-Jerpe et al.^49^ and Fox et al.^56^. The isotope data (δ^15^N and δ^13^C) were pooled and cleaned in the same way as the correlation analysis (above), with the added criterion that only paired δ^15^N and δ^13^C values for both host and symbiont fractions were included. This resulted in sample sizes of n = 18 for *S. pistillata*, n = 14 for *Acropora* sp., and n = 16 for *M. dichotoma*. Following this, we visualised the isotopic niches of each species by plotting the standard ellipse areas of the coral host and symbiont fractions. Next, standard ellipse areas (SEAb) were calculated directly from posterior sampling of the SIBER model, and niche widths were summarised using the posterior mode along with 50% and 95% credible intervals. Thirdly, we used bootstrapped estimates (n = 10,000) of SEAc overlap between the host and symbiont fractions as a proxy for trophic strategy (Conti-Jerpe et al.^49^). In addition, we calculated Layman’s metrics^57^ which serve as useful descriptions of data dispersion, offering insight into trophic diversity.

The software R (version 4.3.2) (R Core Team, 2023) was used to generate figures using packages ‘ggplot2’^87^, ‘ggpubr’^88^, ‘dplyr’^89^, ‘RColorBrewer’^90^, ‘gridExtra’^91^, ‘cowplot’^92^. Likewise, statistics were also computed in R, using packages ‘rstatix’^93^, ‘dunn.test’^94^, ‘tidyverse’^95^, ‘randomForest’^96^, ‘pdp’^97^, ‘SIBER’^98^ and ‘rjags’^99^.

## Supplementary information

Supplementary Information

## Data Availability

All data can be accessed via the public repository Zenodo (Hill et al.^100^ https://doi.org/10.5281/zenodo.17951369), or upon direct request to the author C.E.L. Hill. The isotopic and elemental data shared with Thobor et al.^56^ are available in Zenodo (Hill et al.^101^ https://doi.org/10.5281/zenodo.17849457).
