# Supplementary material for: Coral-associated denitrification is seasonally variable and species-specific
Source: Commun Biol. 2026 Sep 26;9:1259. doi: 10.1038/s42003-026-11026-w (PMC13615964; doi:10.1038/s42003-026-11026-w)
Supplement: Supplementary file 1 [file 42003_2026_11026_MOESM1_ESM.pdf]

Supplementary material

Table S1 Significant relationships identified when comparing seasonal denitrification rates across species (Figure 1a).

Species 1	Species 2	*p* value
Dendrophyllidae indet	*Stylophora pistillata*	0.001
Dendrophyllidae indet	*Acropora* sp.	0.04
Dendrophyllidae indet	*Millepora dichotoma*	0.04

Table S2 Significant relationships identified when comparing denitrification rates across months per species (denoted by letters in Figure 1 b – e).

Species	Group 1	Group 2	Adjusted *p* value
Acropora sp.	April	June	0.03
Acropora sp.	June	December	0.03
Acropora sp.	June	October	0.02
Millepora dichotoma	April	June	0.04
Millepora dichotoma	June	August	0.01
Stylophora pistillata	April	August	0.03
Stylophora pistillata	April	December	0.03
Stylophora pistillata	June	August	0.02
Stylophora pistillata	June	February	0.03
Stylophora pistillata	June	October	0.04
Dendrophylliidae indet	April	December	0.01
Dendrophylliidae indet	June	December	0.005

Table S3 Significant relationships identified when comparing denitrification rates between light and dark incubations per month (denoted by brackets in Figure 1b – e).

Species	Month	Group 1	Group 2	Adjusted *p* value
Stylophora pistillata	April	Dark	Light	0.008
Stylophora pistillata	October	Dark	Light	0.02
Acropora sp.	August	Dark	Light	0.008
Acropora sp.	February	Dark	Light	0.007
Acropora sp.	June	Dark	Light	0.04
Acropora sp.	October	Dark	Light	0.02
Millepora dichotoma	June	Dark	Light	0.03
Millepora dichotoma	October	Dark	Light	0.008
Dendrophylliidae indet	April	Dark	Light	0.008
Dendrophylliidae indet	August	Dark	Light	0.005
Dendrophylliidae indet	February	Dark	Light	0.007
Dendrophylliidae indet	June	Dark	Light	0.02
Dendrophylliidae indet	October	Dark	Light	0.01

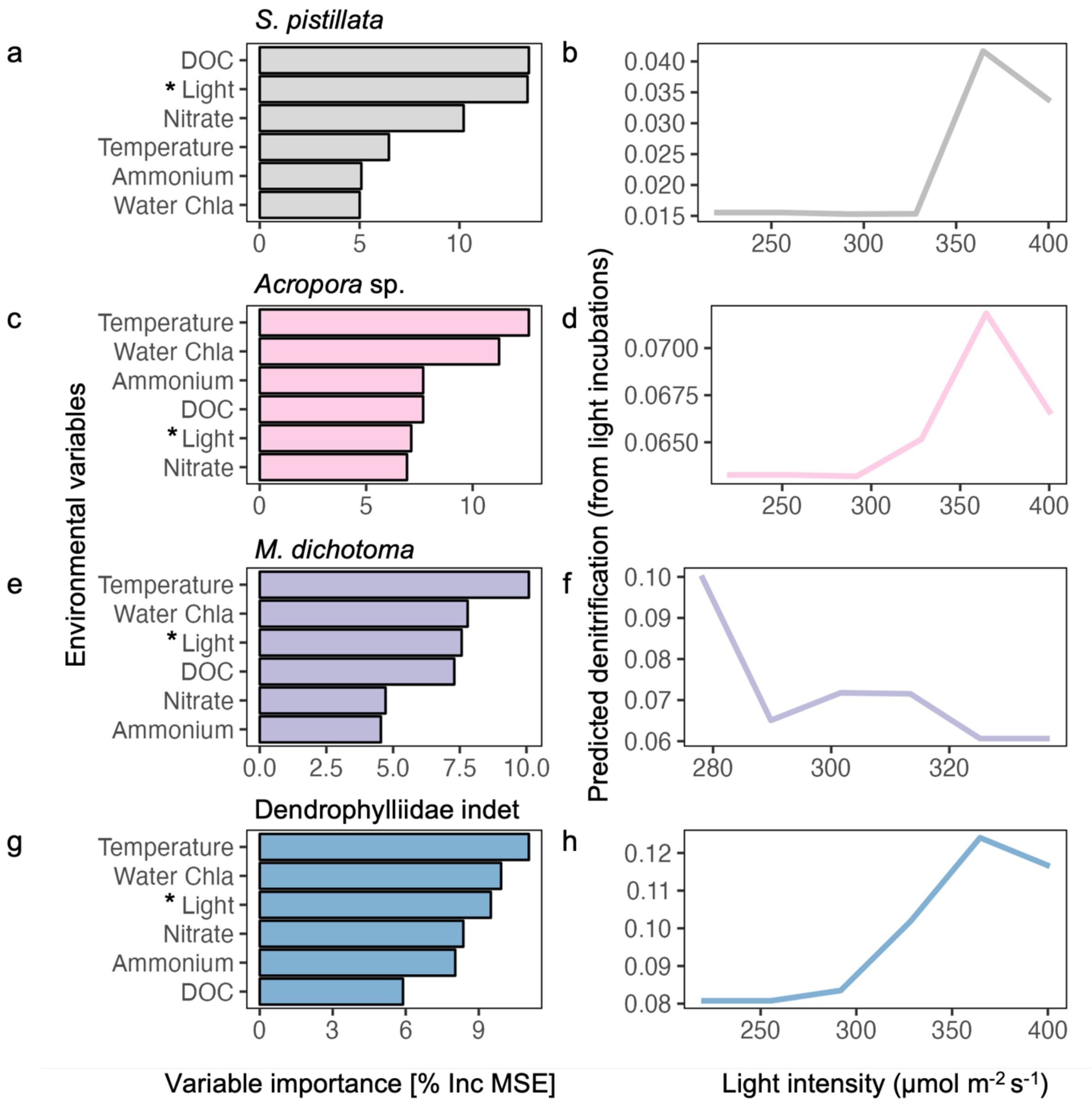

Figure S1 The influence of light intensity (*) on denitrification rates relative to other environmental variables in *Stylophora pistillata* (a – b), *Acropora* sp. (c – d), *Millepora dichotoma* (e - f) and Dendrophylliidae indet (g - h). For each species (each row), the lefthand bar plot shows the ranked importance of five environmental variables in explaining denitrifying variation under light conditions, as identified by random forest analysis. The righthand line graph for each species are partial dependence plots (PDPs), illustrating how light intensity affects denitrification rates. Abbreviations: "DOC" = dissolved organic carbon, "IncMSE" = increase in mean squared error and "Water Chl-a" = water chlorophyll *a*.

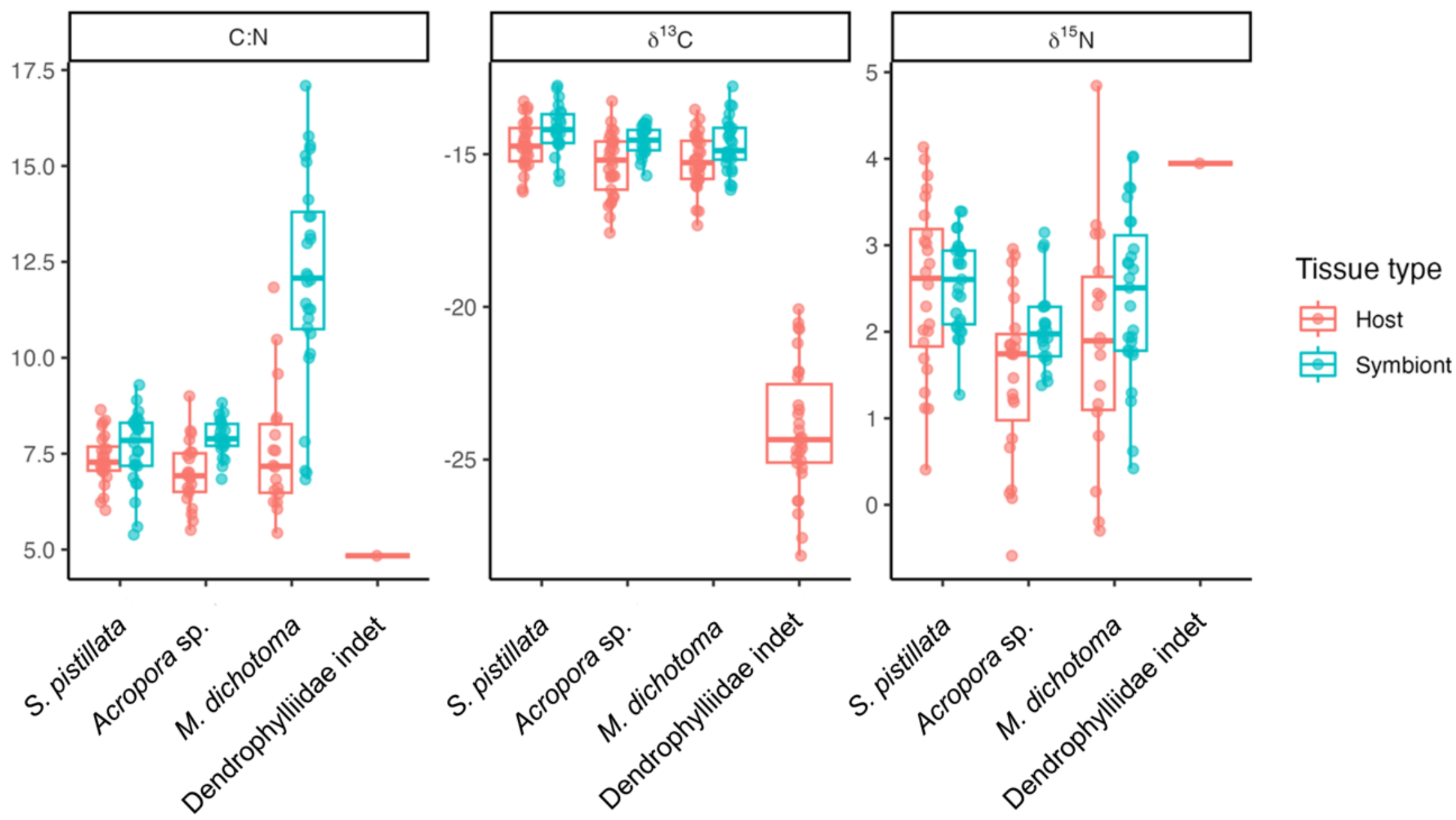

Figure S2 Biogeochemical signatures of each coral *Stylophora pistillata*, *Acropora* sp., *Millepora dichotoma* and Dendrophyllidae indet over the sampling year (April 2022 – February 2023), including elemental C:N, $\delta^{13}C$ and $\delta^{15}N$ split by the host (pink) and symbiont (blue) fractions. For Dendrophylliidae indet, the symbiont fraction is not shown as this coral is azooxanthellate.

Generating acetylene gas

Acetylene gas (1 L) and acetylene-enriched seawater (1 L) were freshly generated for use in the assays the day prior. In brief, 12.5 g of calcium carbide (CaC_2) was reacted with 100 ml of MilliQ water in an Erlenmeyer flask sealed with a rubber stopper. The acetylene gas generated from the reaction, passed via silicone tubing into the first Duran gas washing bottle filled with 1 L of MilliQ water, and then subsequently passed into the second 1 L Duran gas washing bottle filled with 1 L of seawater. Remaining gas then passed via silicone tubing into a gas collection bag (Tedlar 1 L sampling bags with polypropylene valve, RESTEK). The 1 L of acetylene-enriched seawater and 1 L of acetylene gas were used to set up the acetylene assays.

Microsensor technical details

It consisted of a Clark-type microelectrode with a tip size of 50 μm (N_2O-NP-804195, Unisense A/S, Aarhus, Denmark) connected to a high-sensitivity picoampermeter (PA 2000, Unisense

A/S, Aarhus, Denmark). A voltage of - 0.8 V was applied between the cathode and the internal reference anode, whilst N_2O was driven by the external partial pressure to pass through the sensor membrane at the tip (silicone membrane), then it was reduced at the metal cathode surface, while the picoampermeter converted the resulting current to a signal representing the N_2O concentration. Prior to any measurement, the sensor was pre-activated by applying a voltage of -1.3 V for 30 minutes and pre-polarized at -0.8 V for 12 hours until the signal became stable. The sensor was equipped with a front guard made of an ascorbate solution to scavenge incoming O_2 that may interfere with the measuring cathode.

Microsensor method and data normalisation steps

To minimise the signal to noise ratio, the N_2O microsensor was fixed in a stable and stationary position using a clamp. The septa of the gas vials were individually pierced by the microsensor, and once no drift in the sensor signal was observed, 5 replicate readings were recorded in mV and were automatically converted into µmol/L using the calibration curve. Between each sample, the microsensor tip was rinsed with MiliQ water and dried off (Kimwipes, Kimtech Science). Following this, there was a waiting period of variable length (~ 5 – 15 min) while the signal re-stabilised and showed no drift, before the next sample was measured. Gas measurements were subsequently normalised to account for the solubility of N_2O in the MilliQ water of the storage vial and seawater of the beaker, and additionally the volume of the beaker (step by step equations 3-8). Following this, T0 values were subtracted from T12 values to obtain values of N_2O per 12 h. Values were then divided by 12 to be expressed per hour. Following this, the average of five controls were subtracted, to account for background N2O production potentially from microorganisms in the water. As a next step, values were normalised to surface area (cm^{-2}), which was determined by wax dipping the skeleton of the fragment, post-incubation. Values were then converted to N, by multiplying by two and converted to nanomoles. Therefore, denitrification rates were expressed as nmol N cm^{-2} h^{-1}.

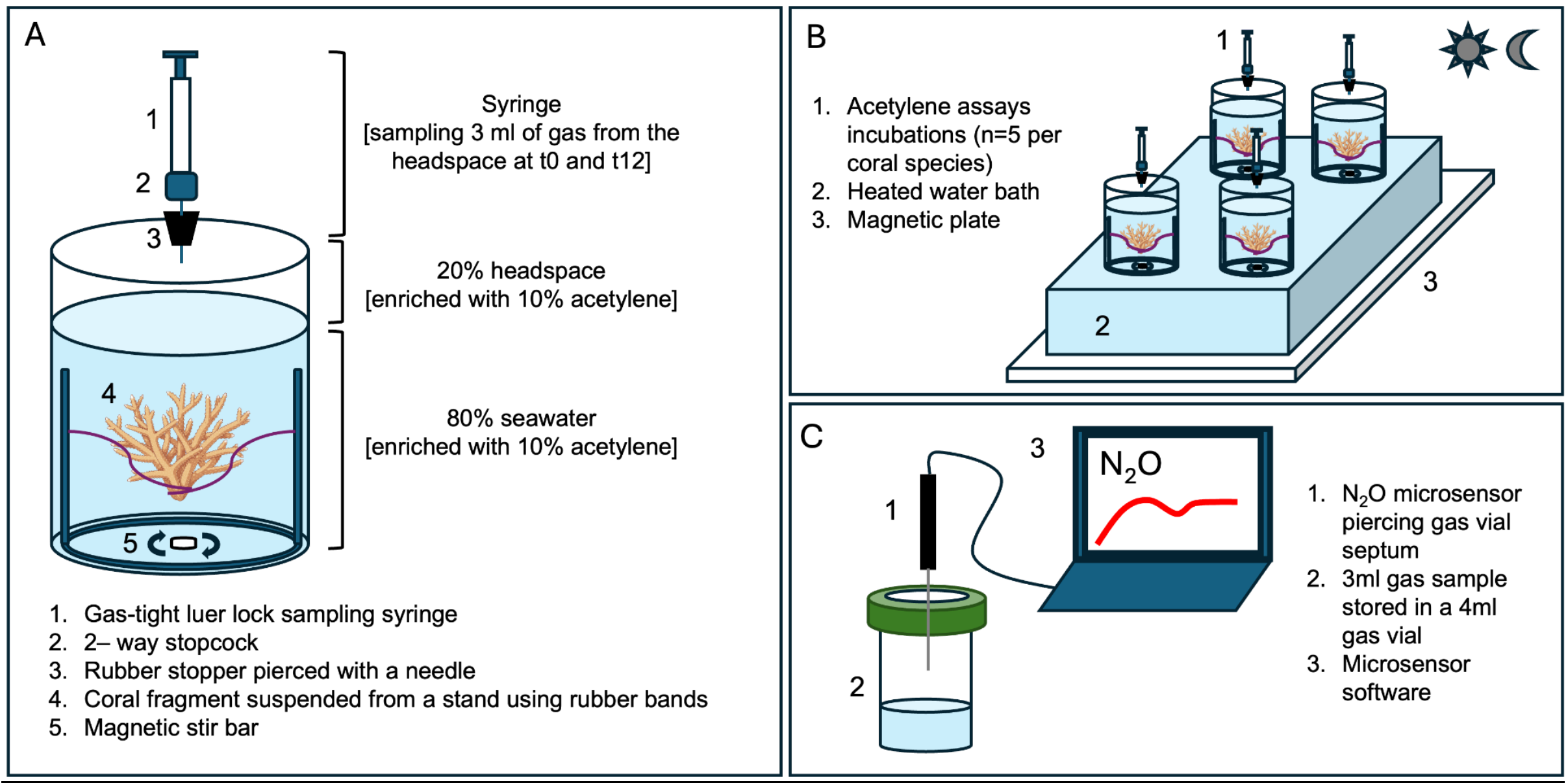

Figure S3 Overview of the quantification of denitrification. A] The acetylene assay setup per coral fragment. Assays were set up in the same manner for controls, just excluding the coral fragment. B] The acetylene assay incubations, performed for 12 hours in the light and 12 hours in the dark. C] Use of a microsensor to measure nitrous oxide (N_2O) of the gas samples generated from the acetylene assay.

Step by step N_2O normalisation equations

[$S^{HS(M)}$] Moles of N_2O in the storage vial headspace (µmol):

$$MV * S^{HS(V)} = S^{HS(M)}$$

[$S^{W(M)}$] Moles of N_2O in the storage vial water: (µmol):

$$MV * BC * S^{W(V)} = S^{W(M)}$$

[$B^{HS(C)}$] Concentration of N_2O in the beaker headspace: (µmol L^{-1}):

$$\frac{S^{HS(M)} + S^{W(M)}}{S^{HS(V)}} = B^{HS(C)}$$

[$B^{HS(M)}$] Moles of N_2O in the beaker headspace (µmol):

$$B^{HS(C)} * B^{HS\,(V)} = B^{HS(M)}$$

[$B^{W(M)}$] Moles of N_2O in the beaker water (µmol):

$$B^{HS(C)} * BC * B^{W\,(V)} = B^{W(M)}$$

[$B^{T(M)}$] Total moles of N_2O in the whole beaker (µmol):

$$B^{HS(M)} + B^{W(M)}$$

Abbreviations used in above equations:

[MV] Microsensor value (µmol L^{-1})
[$S^{HS(V)}$] Storage vial headspace volume (L)
[$S^{HS(M)}$] Storage vial headspace moles (µmol)
[BC] Bunsen solubility coefficient*

**The Bunsen solubility coefficient used varied according to the temperature and salinity. These conditions differed between the storage vial (room temperature of 21 ℃ / salinity of 0) and the beaker (variable temperature dependent on the sampling month and salinity of 39). The coefficient can be determined using a table in* [100].

[$S^{W(V)}$] Storage vial water volume (L)
[$S^{W(M)}$] Storage vial water moles (µmol)
[$B^{HS(C)}$] Beaker headspace concentration (µmol L^{-1})
[$B^{HS(V)}$] Beaker headspace volume (L)
[$B^{HS(M)}$] Beaker headspace moles (µmol)
[$B^{W(V)}$] Beaker water volume (L)
[$B^{W(M)}$] Beaker water moles (µmol)
[$B^{T(M)}$] Beaker total moles (µmol)

Table S4 Light intensities supplied during the light incubations per sampling month. Values were recorded using a Hobo Pendant logger.

**Sampling month**	**Light intensity (photosynthetic active radiation µmol photons m^{-2} s^{-1})**
April 2022	401 ± 20
June 2022	352 ± 17
August 2022	335 ± 17
October 2022	298 ± 21
December 2022	219 ± 18
February 2023	315 ± 7

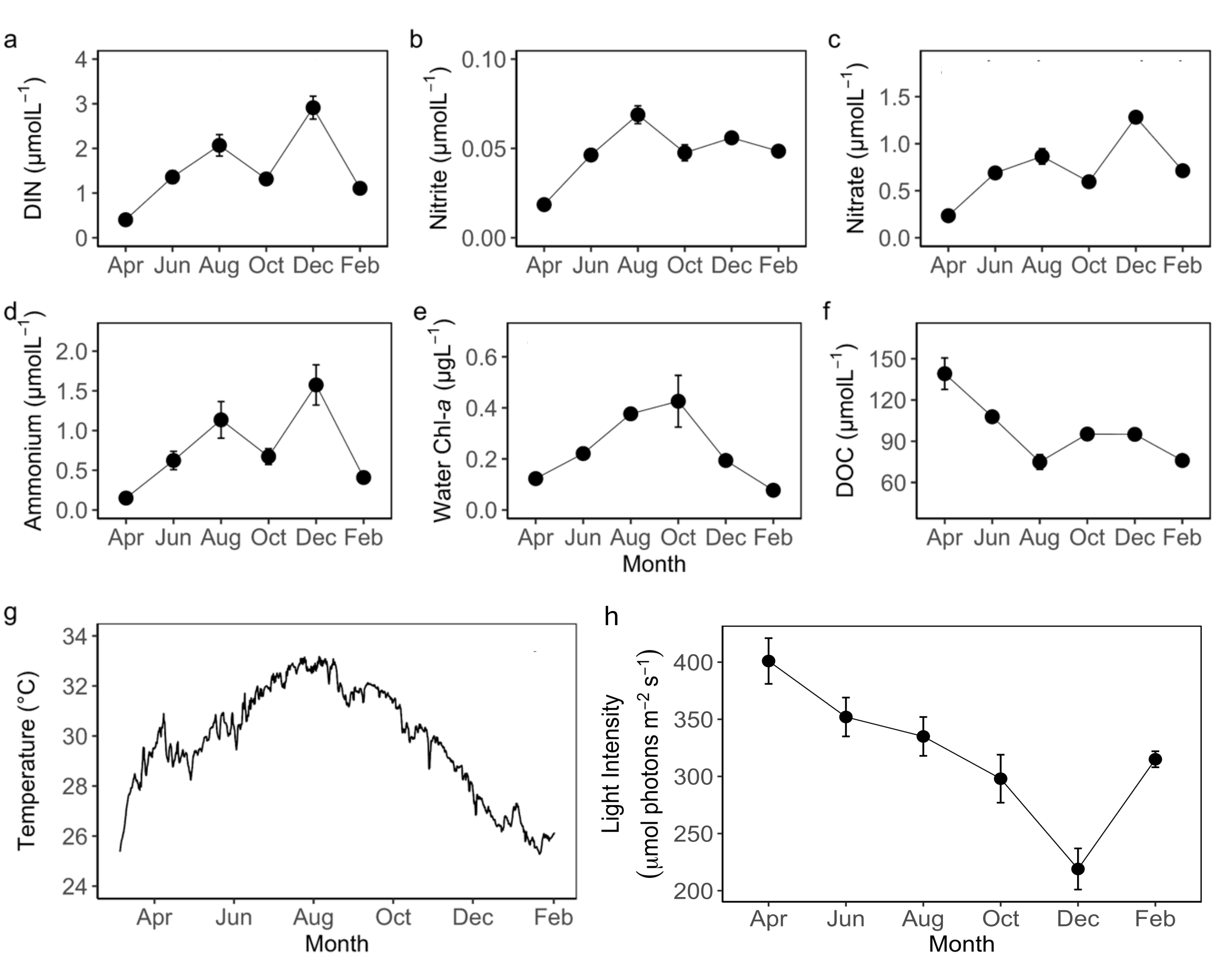

Figure S4 *In situ* environmental parameters monitored throughout the sampling year from April 2022 to February 2023, including average values ± standard error of a) dissolved inorganic nitrogen (DIN), b) nitrite, c) nitrate, d) ammonium e) water chlorophyll *a*, f) dissolved organic carbon (DOC) g) a smooth averaged of temperature and h) light intensity.
